# Correction: Low-Dose Irradiation Affects Expression of Inflammatory Markers in the Heart of ApoE ^-/-^ Mice

**DOI:** 10.1371/journal.pone.0157616

**Published:** 2016-06-09

**Authors:** Daniel Mathias, Ronald E. J. Mitchel, Mirela Barclay, Heather Wyatt, Michelle Bugden, Nicholas D. Priest, Stewart C. Whitman, Markus Scholz, Guido Hildebrandt, Manja Kamprad, Annegret Glasow

## Notice of Republication

This article was republished on June 1, 2016, to correct errors in the in-text references throughout the article. The in-text references did not accurately correspond to the references 14–78 in the Reference list. The authors apologize for the errors. Please download this article again to view the correct version. The originally published, uncorrected article and the republished, corrected article are provided here for reference.

## Supporting Information

S1 File Originally published, uncorrected article. (PDF)

S2 File Republished, corrected article. (PDF)
