# Supplementary material for: Correction: Low-Dose Irradiation Affects Expression of Inflammatory Markers in the Heart of ApoE -/- Mice
Source: PLoS One. 2016 Jun 9;11(6):e0157616. doi: 10.1371/journal.pone.0157616 (PMC4900545; doi:10.1371/journal.pone.0157616)
Supplement: S1 File — (PDF) [file pone.0157616.s001.PDF]

PLOS | ONE

RESEARCH ARTICLE

Low-Dose Irradiation Affects Expression of Inflammatory Markers in the Heart of ApoE $^{-/-}$ Mice

Daniel Mathias[1], Ronald E. J. Mitchel[2], Mirela Barclay[3,4], Heather Wyatt[2], Michelle Bugden[2], Nicholas D. Priest[2], Stewart C. Whitman[3,4†], Markus Scholz[5], Guido Hildebrandt[6], Manja Kamprad[7], Annegret Glasow[1]*

1 Department of Radiation Therapy, University of Leipzig, Leipzig, Germany, **2** Radiological Protection Research and Instrumentation Branch, Canadian Nuclear Laboratories, Chalk River, Ontario, Canada, **3** Departments of Pathology and Laboratory Medicine and Cellular and Molecular Medicine, University of Ottawa, Ottawa, Ontario, Canada, **4** Vascular Biology Group, University of Ottawa Heart Institute, Ottawa, Ontario, Canada, **5** Institute for Medical Informatics, Statistics and Epidemiology, University of Leipzig, Germany, **6** Department of Radiotherapy and Radiation Oncology, University of Rostock, Rostock, Germany, **7** Institute of Clinical Immunology and Transfusion Medicine, University of Leipzig, Leipzig, Germany

† Deceased.
* Annegret.Glasow@medizin.uni-leipzig.de

CrossMark
click for updates

OPEN ACCESS

Citation: Mathias D, Mitchel REJ, Barclay M, Wyatt H, Bugden M, Priest ND, et al. (2015) Low-Dose Irradiation Affects Expression of Inflammatory Markers in the Heart of ApoE $^{-/-}$ Mice. PLoS ONE 10(3): e0119661. doi:10.1371/journal.pone.0119661

Academic Editor: Liwu Li, Virginia Polytechnic Institute and State University, UNITED STATES

Received: July 2, 2014

Accepted: January 21, 2015

Published: March 23, 2015

Copyright: © 2015 Mathias et al. This is an open access article distributed under the terms of the Creative Commons Attribution License, which permits unrestricted use, distribution, and reproduction in any medium, provided the original author and source are credited.

Data Availability Statement: All relevant data are within the paper.

Funding: The project was funded by European commission grant FI6R036465, NOTE. The funders had no role in study design, data collection and analysis, decision to publish, or preparation of the manuscript.

Competing Interests: The authors have declared that no competing interests exist.

Abstract

Epidemiological studies indicate long-term risks of ionizing radiation on the heart, even at moderate doses. In this study, we investigated the inflammatory, thrombotic and fibrotic late responses of the heart after low-dose irradiation (IR) with specific emphasize on the dose rate. Hypercholesterolemic ApoE-deficient mice were sacrificed 3 and 6 months after total body irradiation (TBI) with 0.025, 0.05, 0.1, 0.5 or 2 Gy at low (1 mGy/min) or high dose rate (150 mGy/min). The expression of inflammatory and thrombotic markers was quantified in frozen heart sections (CD31, E-selectin, thrombomodulin, ICAM-1, VCAM-1, collagen IV, Thy-1, and CD45) and in plasma samples (IL6, KC, MCP-1, TNFα, INFγ, IL-1β, TGFβ, INFγ, IL-10, sICAM-1, sE-selectin, sVCAM-1 and fibrinogen) by fluorescence analysis and ELISA. We found that even very low irradiation doses induced adaptive late responses, such as increases of capillary density and changes in collagen IV and Thy-1 levels indicating compensatory regulation. Slight decreases of ICAM-1 levels and reduction of Thy 1 expression at 0.025–0.5 Gy indicate anti-inflammatory effects, whereas at the highest dose (2 Gy) increased VCAM-1 levels on the endocardium may represent a switch to a pro-inflammatory response. Plasma samples partially confirmed this pattern, showing a decrease of proinflammatory markers (sVCAM, sICAM) at 0.025–2.0 Gy. In contrast, an enhancement of MCP-1, TNFα and fibrinogen at 0.05–2.0 Gy indicated a proinflammatory and prothrombotic systemic response. Multivariate analysis also revealed significant age-dependent increases (KC, MCP-1, fibrinogen) and decreases (sICAM, sVCAM, sE-selectin) of plasma markers. This paper represents local and systemic effects of low-dose irradiation, including also age- and dose rate-dependent responses in the ApoE $^{-/-}$ mouse model. These insights

in the multiple inflammatory/thrombotic effects caused by low-dose irradiation might facilitate an individual evaluation and intervention of radiation related, long-term side effects but also give important implications for low dose anti-inflammatory radiotherapy.

Introduction

Epidemiological studies of Japanese atomic bomb survivors [1–3] and medically exposed groups [4,5] indicate long-term risks of non-cancer health effects, such as cerebrovascular and, even more commonly, cardiovascular diseases after exposure to ionizing radiation at moderate doses. In occupational exposed workers, a significant association between radiation and atherosclerotic heart disease was observed even at very low doses of ~30 mSv [6].

Radiation-induced heart diseases (RIHD), after irradiation of mediastinal structures, became obvious in the late 1960s, when long-term survival of Hodgkin's disease patients improved and radiation therapy (RT) was increasingly applied to a broad range of malignant tumors such as breast cancer [7]. Although RIHD affects any cardiac structure [8], with pericardial involvement being the most common at high doses [9], blood vessels are important targets of irradiation. Tissue damage, mediated by vascular injury, is thought to be one of the most consistent pathogenetic mechanisms in delayed radiation injury. Thereby, endothelial cells in the microvasculature represent the most radiation-vulnerable elements [10].

Vascular injuries caused by high irradiation doses are well studied and include acute vasculitis with heterophil invasion, endothelial cell (EC) swelling, capillary loss, and activation of coagulatory mechanisms, along with local ischemia and fibrosis [11,12]. Estimation of health effects at low irradiation doses, however, can not be drawn by extrapolation of high-dose effects. Studies on aortic lesions and serum cholesterol levels after single low-dose exposures revealed that radiation-induced effects were distinctly non-linear with doses. Thereby, the dose rate turned out to be an important parameter with low dose rates showing rather protective, but high dose rates exerting protective as well as detrimental effects [13]. Detailed experimental data concerning low-dose radiation effects on the heart microvasculature or on systemic plasma parameters at late time points are missing so far.

We and others have shown that irradiation triggers inflammatory, thrombotic and fibrotic processes in the murine vasculature similar to epidemiological and clinical findings in humans [13–16].

Here we studied for the first time late effects of total body irradiation (TBI) at very low doses (0.025–2.0 Gy) on the microvasculature of the heart and on plasma markers, with particular emphasis on different dose rates (1.0 mGy/min and 150 mGy/min). To gain more insight into the long-term dose response relationship at low-dose exposures, we analyzed 8 relevant markers (CD31, E-selectin, thrombomodulin, ICAM-1, VCAM-1, collagen IV, Thy-1, CD45) by quantitative immunofluorescence and 11 systemic plasma markers (IL6, KC, MCP-1, TNF, TGF, INFγ, IL-10, sICAM-1, sE-selectin, sVCAM-1 and fibrinogen) by commercial ELISA or cytometric bead array 3 and 5 months after TBI of Apoprotein $E^{-/-}$ ($ApoE^{-/-}$) mice. $ApoE^{-/-}$ mice have elevated plasma cholesterol levels and spontaneously develop atherosclerosis, a multistep process driven by inflammation [17]. Recent data indicate that $ApoE^{-/-}$ mice represent a more sensitive model than the corresponding wild-type mice [14,18], possibly enabling the detection of even very small inflammatory or thrombotic processes, expected at low irradiation doses.

The pathogenetic mechanisms of radiation-induced vascular effects involve the acute induction of reactive oxygen intermediates [19], which subsequently activate intracellular signal cascades, cytokines, transcription factors and adhesion molecules expressed on ECs, fibroblasts and leukocytes. Chronic free radical production and oxidative stress, possibly linked by sustained upregulation of NF-$_{\kappa}$B transcription factor towards chronic inflammation, are increasingly implicated in the radiation-induced late tissue injury [20]. In a multistep cascade, upregulated selectin receptors on ECs may then capture free leukocytes and mediate their rolling and transient adhesion along the vessel wall. Thereafter, adhesion molecules, such as ICAM-1, VCAM-1 and Thy-1, interact with specific integrins on the leukocytes ensuring their firm adhesion to ECs. During diapedesis, leukocytes transmigrate through endothelial tight junctions, which is mediated mainly by CD31 [21–25].

CD45, a pan leukocyte marker, has been used here to evaluate the presence of leukocytes in heart tissue with and without IR. IR-induced disturbances in the (anti-)coagulant system may lead to endothelial damage and chronic organ dysfunction. Thrombomodulin has been included in this study, for its function as a crucial player in the maintenance of the haemostatic balance. It is involved in vascular thromboresistance and anticoagulant homeostasis [26–28] but may also affect the expression of adhesion molecules [29]. During radiotherapy, dose-dependent increases of plasma TM in cancer patients were observed and it has been suggested as a marker of radiation-induced endothelial cell injury [30].

Collagen IV, expressed by endothelial and epithelial cells, occurs in the basement membrane, which encloses every capillary, and is involved in blood vessel function, cellular supply and cell signal cascades [31]. It is used here as an indicator of fibrosis [32–34] and has been reported to change after irradiation [35].

In Japanese atomic bomb survivors and in patients with postoperative radiotherapy, considerable systemic effects of ionizing radiation have been documented. Elevated plasma cytokine levels, namely IL6 and TNFα, were indicative for a persistent inflammatory status [36,37]. In contrast, low-dose radiotherapy is known to exert anti-inflammatory effects, involving the TGFβ-induced downregulation of leukocyte/endothelial cell adhesion [38–40].

To study systemic effects of radiation, we measured both, established proinflammatory (TNFα, KC, MCP-1, IL6, INFγ) and anti-inflammatory (TGFβ, IL10) cytokines [37]. Plasma levels of soluble adhesion molecules (sICAM, sVCAM, sE-selectin) have been proven to be useful markers for the assessment of inflammatory processes involving activation and injury of the endothelium in various conditions, such as stem cell transplantation-related complications, radiation pneumonitis and coronary disease [41–43]. Fibrinogen is included in this study as systemic biomarker for inflammatory/thrombotic disease [44]. Significant enhancements of plasma fibrinogen levels have been observed after WBI in rats [45], in previously irradiated Hodgkin lymphoma (HL) survivors [46], and in subjects with cardiovascular disease compared to those without [47].

Materials and Methods

Mice, irradiation, tissue preparation

ApoE$^{-/-}$ (B6.129P2-ApoE$^{tml/Unc}$/J) female mice, homozygous null for a functional ApoE gene (in C57BL/6J background) were transferred at 6 weeks from Jackson Laboratory (Bar Harbor, ME, USA) to the Chalk River Laboratories (Ontario, Canada). Mice were provided with *ad libitum* reverse osmosis deionized, UV-light sterilized water and Charles River Rodent Chow no. 5075, autoclaved, normal low-fat (4.5%) diet (Charles River Canada, St. Constant, Quebec). Female ApoE$^{-/-}$ mice were exposed to ^{60}Co γ-radiation (0.025, 0.05, 0.1, 0.5 or 2 Gy) at 8 weeks of age. Irradiation was performed at either low dose rate (LDR, 1 mGy/min) from an open beam

source (GammaBeam 150, Canadian Nuclear Laboratories) on unrestrained mice in their cages or at high dose rate (HDR, 0.15 Gy/min) in an enclosed irradiator (GammaCell 200, Canadian Nuclear Laboratories). Animals received their designated dose as a single exposure except those receiving 0.5 or 2.0 Gy at low dose rate. Those two groups received 100 mGy per day at 1 mGy/min for 5 consecutive days for the 0.5 Gy group or for 5 days/week for 4 weeks for the 2.0 Gy group. Control (0 Gy) mice were handled in the same manner as exposed mice but placed in a shielded space during the exposure of the test group, with more details described previously (12). Housing, handling and experimental procedures were conducted in accordance with the guidelines of the Canadian Council on Animal Care (Membership Number KG05) and with the preapproval of the local Atomic Energy Canada Laboratories (now Canadian Nuclear Laboratories), Animal Care Committee, Chalk River Laboratories Animal Care Committee (Protocol 06-02).

Mice were shipped to the University of Ottawa Heart Institute and euthanized with an intraperitoneal injection of sodium pentobarbital prior to blood and tissue collections 3 or 6 months after exposure (at 5 or 8 months of age). Shock-frozen hearts were embedded in O.C.T. tissue-tek (Sakura Finetek, Torrance, USA) and sent to the University of Leipzig. Cryosections (7 µm) were prepared and stored at −80°C (samples detailed in Table 1). EDTA plasma samples (detailed in Table 1) were sent to the University of Leipzig. There are more plasma samples than tissue samples, because a part of the tissues was destroyed (defrosted) during transport.

Staining

Sections from two regions of the heart (heart base and apex) were examined. Sections of each subgroup (dose rate, age) were stained together with samples from sham-irradiated control animals and negative staining controls.

Specimens were stained with hematoxylin/eosin (HE) and sirius red following standard protocols. For specific immune fluorescence staining, sections were fixed in ice-cold ethanol/acetone, 50/50, for 10 min. Specimens were then washed with PBS (3 min) and incubated in blocking solution (PBS, bovine serum albumin 2%) for 20 min. Primary antibodies or isotype specific Ig (negative staining controls) were applied for 1 h diluted in blocking solution.

Table 1. Samples.

Dose [Gy]	Dose rate	Age at time of sacrifice	Tissue samples	Plasma samples
0	-	2 months	4	7
0	sham irradiation	5 months	3	7
0	sham irradiation	8 months	5	7
0.025	LDR	5 months/8 month	4 of either age	7 of either age
0.05	LDR	5 months/8 month	4 of either age	7 of either age
0.1	LDR	5 months/8 month	4 of either age	7 of either age
0.5	LDR	5 months/8 month	4 of either age	7 of either age
2	LDR	5 months/8 month	4 of either age	7 of either age
0.025	HDR	5 months/8 month	4 of either age	7 of either age
0.05	HDR	5 months/8 month	4 of either age	7 of either age
0.1	HDR	5 months/8 month	4 of either age	7 of either age
0.5	HDR	5 months/8 month	4 of either age	7 of either age
2	HDR	5 months/8 month	4 of either age	7 of either age

HDR: high dose rate, LDR: low dose rate

doi:10.1371/journal.pone.0119661.t001

Table 2. Antibodies.

Antibodies	Species	Source
**Primary antibodies**		
anti-mouse CD31 (IgG2αK)	rat	eBioscience, San Diego (USA)
anti-mouse E-Selectin	chicken	R&D Systems, Minneapolis (USA)
anti-mouse Thrombomodulin	goat	Santa Cruz, Santa Cruz (USA)
anti-mouse ICAM-1	goat	R&D Systems, Minneapolis (USA)
anti-mouse VCAM-1 (IgG2αK)	rat	eBioscience, San Diego (USA)
anti-mouse Collagen IV A3	goat	Santa Cruz, Santa Cruz (USA)
anti-mouse Thy-1 (IgG1α)	rat	Abcam Inc., Cambride (UK)
anti-mouse CD45	goat	R&D Systems, Minneapolis (USA)
**Secondary antibodies**		
anti-chicken Cy 5	donkey	Jackson Immuno, West Grove (USA)
anti-rat FITC	donkey	Jackson Immuno, West Grove (USA)
anti-rat Cy 5	donkey	Jackson Immuno, West Grove (USA)
anti-goat Cy 5	donkey	Jackson Immuno, West Grove (USA)
**Control IgG**		
IgGY chicken	chicken	R&D Systems, Minneapolis (USA)
IgG1α rat	rat	R&D Systems, Minneapolis (USA)
IgG2α rat	rat	eBioscience, San Diego (USA)
IgG goat	goat	Santa Cruz, Santa Cruz (USA)

doi:10.1371/journal.pone.0119661.t002

Specimens were washed again, and corresponding, fluorescence-labeled, secondary antibodies were added (Table 2). After 45 min, specimens were rinsed again in PBS and incubated with DAPI nuclear stain for 5 min (Sigma Aldrich, Saint Louis, USA). Sections were embedded in Mowiol 4-88/DABCO (Carl Roth GmbH, Karlsruhe, Germany). Lectin-stained heart sections (fluroescein lycopersicon esculentum tomato lectin, FL-1171, Vector laboratories) were kindly provided by Fiona Stewart's laboratory, NKI (The Netherlands Cancer Institute).

Analysis

Quantitative assessment of fluorescence staining: Preview areas of complete myocardial cross sections were generated using 50x magnification and a semiautomatic immunofluorescence microscope (Zeiss AxioImager Z.1, Carl Zeiss MicroImaging, Göttingen, Germany) and associated microscopic software (Tissue Fax, Tissue Gnostics, Vienna, Austria). Regions of interest (ROI) were marked and scanned using 50x magnification (Fig. 1A). Specific expression patterns of CD31, intercellular adhesion molecule-1 (ICAM-1), thrombomodulin, thymocyte antigen-1 (Thy-1) and E-selectin, were analyzed only in areas with cross sections of capillaries around the left ventricle using 200x magnification. Areas with larger vessels, cracks or longitudinal sections of capillaries were excluded from fields of view (FOV). All FOVs within a ROI were analyzed using semiautomatic cell detection software Tissue Quest (Tissue Gnostics, Vienna, Austria). The number of stained capillaries was normalized on the nuclei count based to DAPI staining (approx. 1000–8000 per ROI, Fig. 1B, C).

The presence of collagen IV and CD45 was examined in the whole myocardium using 5–10 complete FOVs for analysis and were normalized on the area of the analyzed tissue. For the vascular adhesion molecule-1 (VCAM-1), examined in the endocardium, only fluorescence intensity was measured.

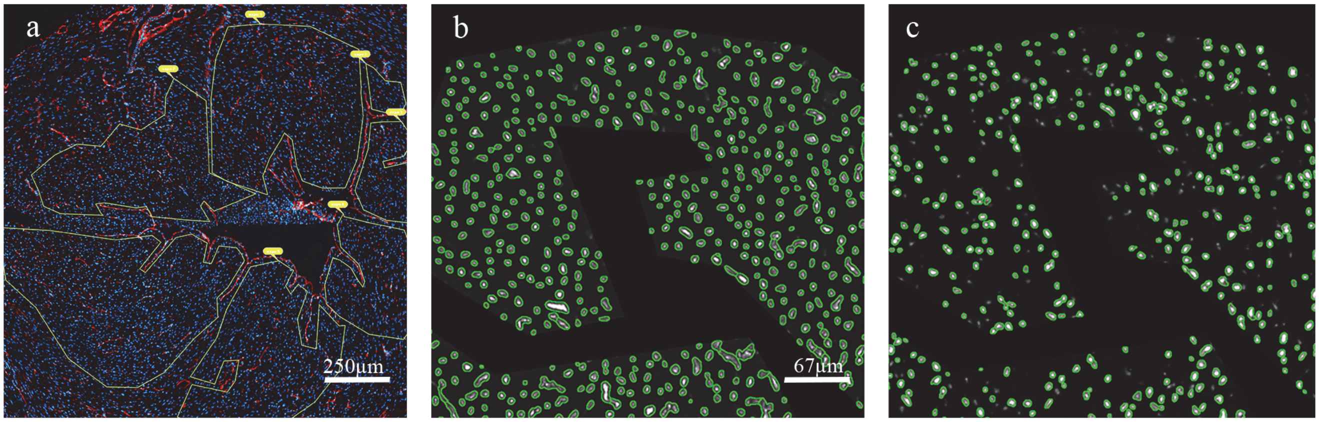

Fig 1. Quantitative analysis of staining by Tissue Fax/Quest. a) Regions of interest (ROI) with cross-sectioned capillaries only, were manually marked and fields of view (FOV) were scanned. Blue = nuclei stained with DAPI, red = capillaries stained with CD31, yellow = ROI, **b)** DAPI-stained nuclei are counted as reference parameter and represent the whole cell count within the FOV, **c**) CD31-stained events are counted in corresponding areas.

doi:10.1371/journal.pone.0119661.g001

EDTA plasma samples (7 samples per group) were analyzed for systemic markers of inflammation. Interleukin-6 (IL6), interleukin-10 (IL10), chemokine KC, macrophage chemoattractant protein-1 (MCP-1), tumor necrosis factor alpha (TNF), interferon (INF) and interleukin-1beta (IL-1), were measured by cytometric bead array (CBA, BD Biosciences) using the mouse inflammation kit (BD Biosiences). ELISA measurements were conducted in diluted plasma samples for soluble adhesion molecules sICAM-1 (1:250, EMICAM12e, Perbio Science), sE-selectin (1:50, MES00, R&D), sVCAM-1 (1:250, MVC00, R&D) fibrinogen (1:10.000, EMF2040-AS, BioCAT) and tumor growth factor beta, TGF (1:36, MC100B, R&D) at 450/690 nm (Spectrafluor plus, TECAN).

Statistics

For CD31, CD45, ICAM-1, thrombomodulin, Thy-1, and E-selectin, the following parameters were analyzed: events, representing the number of positive cells or capillaries (single event represents a single cell), mean fluorescence intensity (mFI), representing the expression level per event, and mean area, representing the size of the event. For VCAM-1, only the mFI was measured.

Effects were analyzed 1) for the main variable: dose and 2) for the covariates: dose rate, age and region. First, main variables and covariates were analyzed univariately by linear mixed models accounting for repeated measurements in animals and staining bath as random factors. In case of univariate significance of covariates, the effect of the irradiation dose was also analyzed multivariately by adjusting for significant covariates (dose rate, age and region). To estimate the effect of the age groups, a mixed-model analysis on non-radiated animals was performed, considering repeated measurements and staining bath as random factors. In addition, subgroup analyses were performed for age and dose rates using ANOVA. All calculations were performed using the statistical software package SAS 9.1.3. (2002–2003, SAS Institute Inc., Cary, NC, USA). Power analysis: Experimental design allows detecting differences of 2.3 SD between 0 Gy and 2 Gy group with 80% power and 5% significance using 4 tissue samples per group. For plasma levels, 7 plasma samples per group allow to detect differences of 1.6 SD. Sample size was calculated with PASS 2008, Version 08.05. (Hintze J. NCSS LLC, Kaysville, Utah). For correlation analysis Kendalls tau correlation was used (R, http://www.R-project.org). Statistical significance is

indicated by asterisk symbols with p-value ≤ 0.05: *, $p \leq 0.01$: **, $p \leq 0.001$: ***, if not otherwise indicated.

Results

Irradiation-induced effects on heart tissue

Immunofluorescence staining. Effects of irradiation have been analyzed on eight inflammatory/thrombotic markers (CD31, E-selectin, thrombomodulin, ICAM-1, VCAM-1, collagen IV, Thy-1, CD45). Results are presented as relative values compared to non-irradiated control animals. Data represent the relative cell counts of marker positive ($^{+ve}$) cells normalized to 1000 nuclei. In the case of endothelial cells, a single positive cell (event) corresponds to one capillary (cross-sectioned).

Response towards irradiation was found to be independent of dose rate (high or low), region (heart base and apex), and time (3 and 6 months after irradiation). No differential effects could be detected by subgroup analysis, except for collagen IV. Therefore, data are given as the mean of all subgroups (HDR and LDR, time, base and apex), except for collagen IV, where time-specific data are shown.

Analyses of mean area (event size) and evaluation of the mean fluorescence intensity (expression levels per event) revealed no significant changes for any of the markers, except for VCAM (only VCAM data are shown). Therefore, we conclude that there are neither significant radiation-induced changes in cell/capillary size nor in the size of collagen deposits. From our data, we may also exclude a significant translocation of markers from the nucleus to the outer membrane as a radiation response, because this would have resulted in a change of the mean area.

Radiation dose-specific effects on event (cell/capillary) counts have been observed for most of the markers and are presented below.

Lectin/CD31. CD31 expression varies in the vasculature depending on size and localization of the vessels. By consecutive serial staining, we confirmed strict colocalization of lectin, an endothelial-specific glycoprotein, and CD31 immunoreactivity on microvascular endothelial cells (Fig. 2). Thus CD31^{+ve} cell count can be used as a marker for capillary cell count in the heart.

Specific staining of endothelial markers is demonstrated in fluorescence images showing positive fluorescence signals on capillaries, endocard (VCAM only) and small vessels (Fig. 3).

CD31/E-selectin and thrombomodulin. These three markers showed similar responses to irradiation and, therefore, they are presented together (Fig. 4A). CD31 staining showed a slight dose-dependent increase of capillary numbers. Significance was reached after 2 Gy IR compared with sham-irradiated controls. E-selectin and thrombomodulin immunoreactivity followed this pattern. These findings indicate that the newly built capillaries express E-selectin and thrombomodulin protein (Fig. 4A). The size of capillaries did not change (data not shown). **ICAM-1/Thy-1 and CD45:** ICAM cell counts also increased slightly at 2 Gy, although not reaching significance. In contrast, low doses caused a slight decrease of ICAM-1^{+ve} cells. Thy-1, another adhesion molecule expressed on endothelial cells but also on fibroblasts, showed a similar decrease of immunoreactive cell counts. A significant effect was seen beginning at very low doses of 0.025 and continuing up to 0.5 Gy. CD45^{+ve} cell counts were only slightly reduced at low doses (Fig. 4B). **VCAM-1** was expressed only at very low levels on capillaries, which did not allow for quantification. Therefore, we evaluated VCAM response after low-dose irradiation on the endocardium, which had high expression levels. Mean fluorescence intensities showed a dose-dependent enhancement of VCAM expression. Significant increases were found after 2 Gy IR (Fig. 4C). For the lack of an appropriate reference

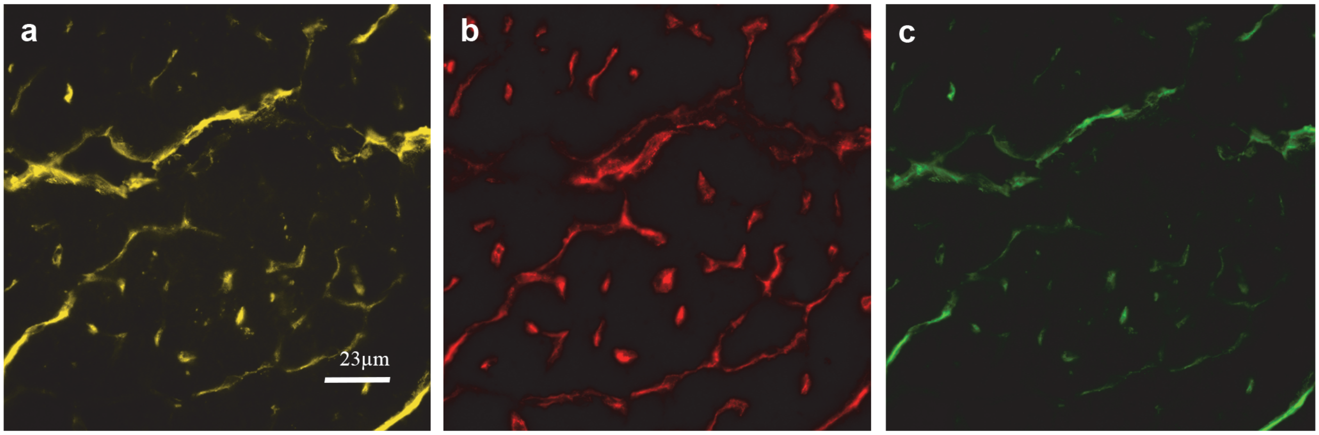

Fig 2. Detection of CD31^{+ve} and Lectin^{+ve} vessels in serial sections. a) Lectin (yellow), **b)** serial section (7 µm apart) stained for CD31 (red), **c)** Matching areas are indicated in green (artificial colours).

doi:10.1371/journal.pone.0119661.g002

parameter, such as nuclei count, analysis of VCAM-1^{+ve} cell count (events) could not be conducted. No changes of VCAM-1^{+ve} total areas were observed. **Collagen IV^{+ve}** cell counts in 5-months-old animals (3 months after irradiation) increased significantly even at the lowest dose

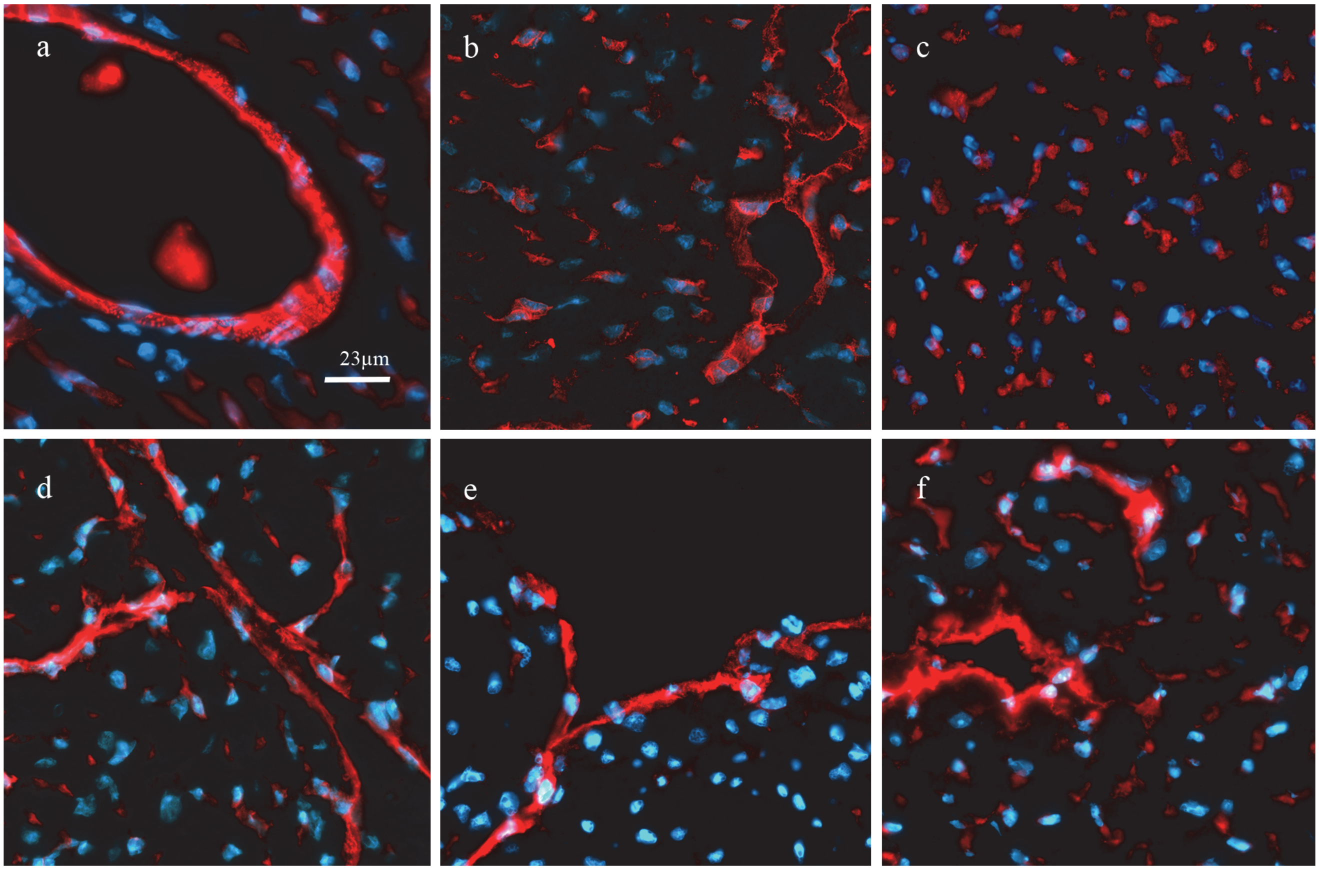

Fig 3. Immunofluorescence staining of endothelial markers. Specific expression of endothelial proteins is shown in red (artificial colour) and nuclei counterstaining in blue (DAPI). CD31 **a)**, E-selectin **b)**, thrombomodulin **c)**, ICAM-1 **d)**, VCAM-1 **e)** and, Thy-1 **f)** were expressed on capillaries, small vessels (arterioles, venules) and endocard. Staining was quantitatively assessed only on capillaries, except for VCAM, which was evaluated on the endocard only.

doi:10.1371/journal.pone.0119661.g003

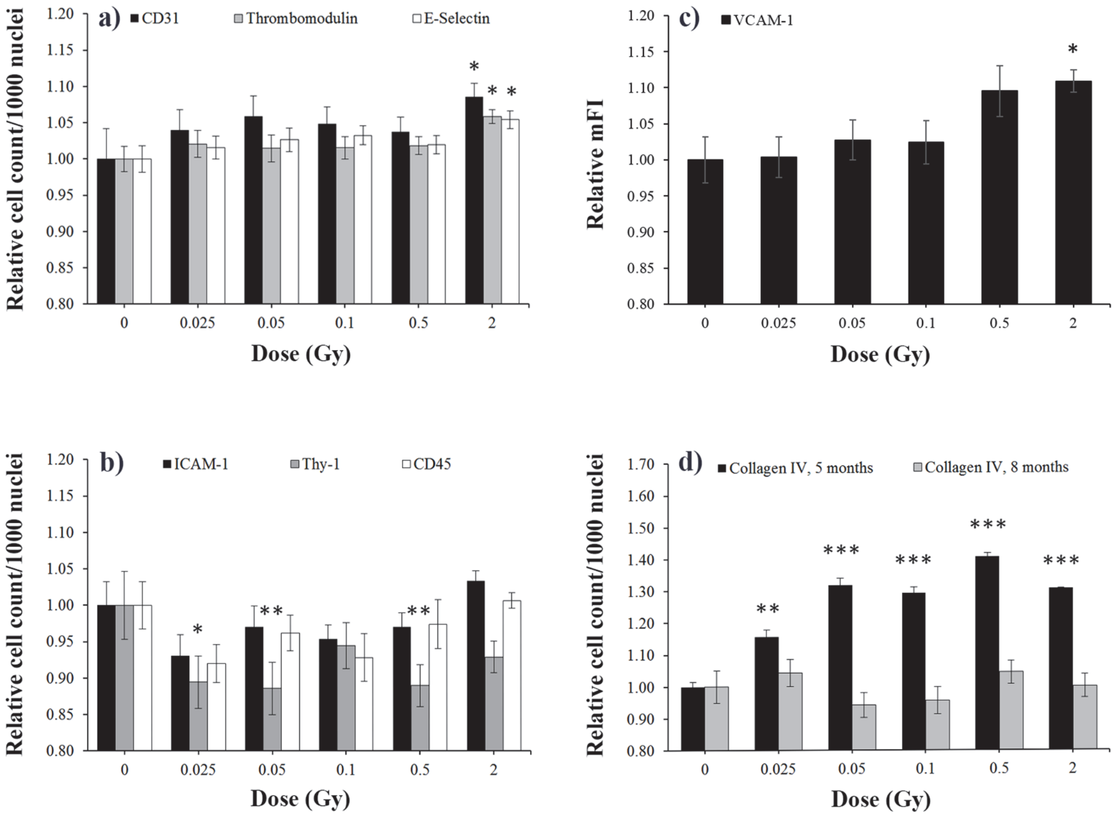

Fig 4. Dose-specific effects on tissue markers. Quantification of immunofluorescence stainings is presented in panel **a)** for CD31, thrombomodulin, E-selectin; **b)** ICAM-1, Thy-1, CD45; **c)** for VCAM, and **d)** for collagen IV. The number of specifically stained cells per 1000 nuclei around the left ventricle of the heart was normalized on 0 Gy values (= 1). Only for VCAM-1 **c)**, relative values of mean fluorescence intensity (mFI) from endocard stainings are presented. Except for collagen IV **d)**, where age-specific data are shown, data are given as mean of all subgroups (HDR and LDR, at 5 and 8 months, corresponding to 3 and 6 months after irradiation, base and apex) ± SEM, n = 16. Asterisks indicate p-values ≤ 0.05: *, p ≤ 0.01: **, p ≤ 0.001: ***.

doi:10.1371/journal.pone.0119661.g004

of 0.025 Gy, with 0.5 Gy dose exerting the strongest effect. In contrast, analysis of 8-months-old animals (6 months after irradiation) revealed only weak changes (Fig. 4D). The mean area did not change, indicating that irradiation has no effect on the size of the collagen deposits (data not shown).

Histological findings. HE and sirius red stainings were performed on all animals to get an overview of irradiation-induced morphologic alterations.

One animal, irradiated with 0.5 Gy (LDR, 5 months group), showed a distinct, sub-endocardial region with disseminated lipocytes, enclosed by disorganized collagenous strands. Immunofluorescence staining revealed an accumulation of CD45^{+ve} and of collagen IV^{+ve} cells (Fig. 5A-C). Four animals, irradiated with 2 Gy (LDR, 8 months group), showed broad fibrotic strands crossing the right ventricle, but no accumulation of leukocytes or collagen IV^{+ve} cells. These findings have been observed in 2 different cross sections of approx. 300 μm distance (Fig. 5 D-F). None of the sham-irradiated control animals showed similar changes.

Irradiation-induced effects in plasma samples

Effects of irradiation have been analyzed on eleven inflammatory/thrombotic plasma markers (IL6, KC, MCP-1, TNF, TGF, INF, IL-10, sICAM-1, sE-selectin, sVCAM-1 and fibrinogen) 3 and 6 months after irradiation at HDR and LDR. Statistical analysis revealed subgroup-specific responses. Therefore values of plasma markers 3 and 6 months after irradiation at HDR and

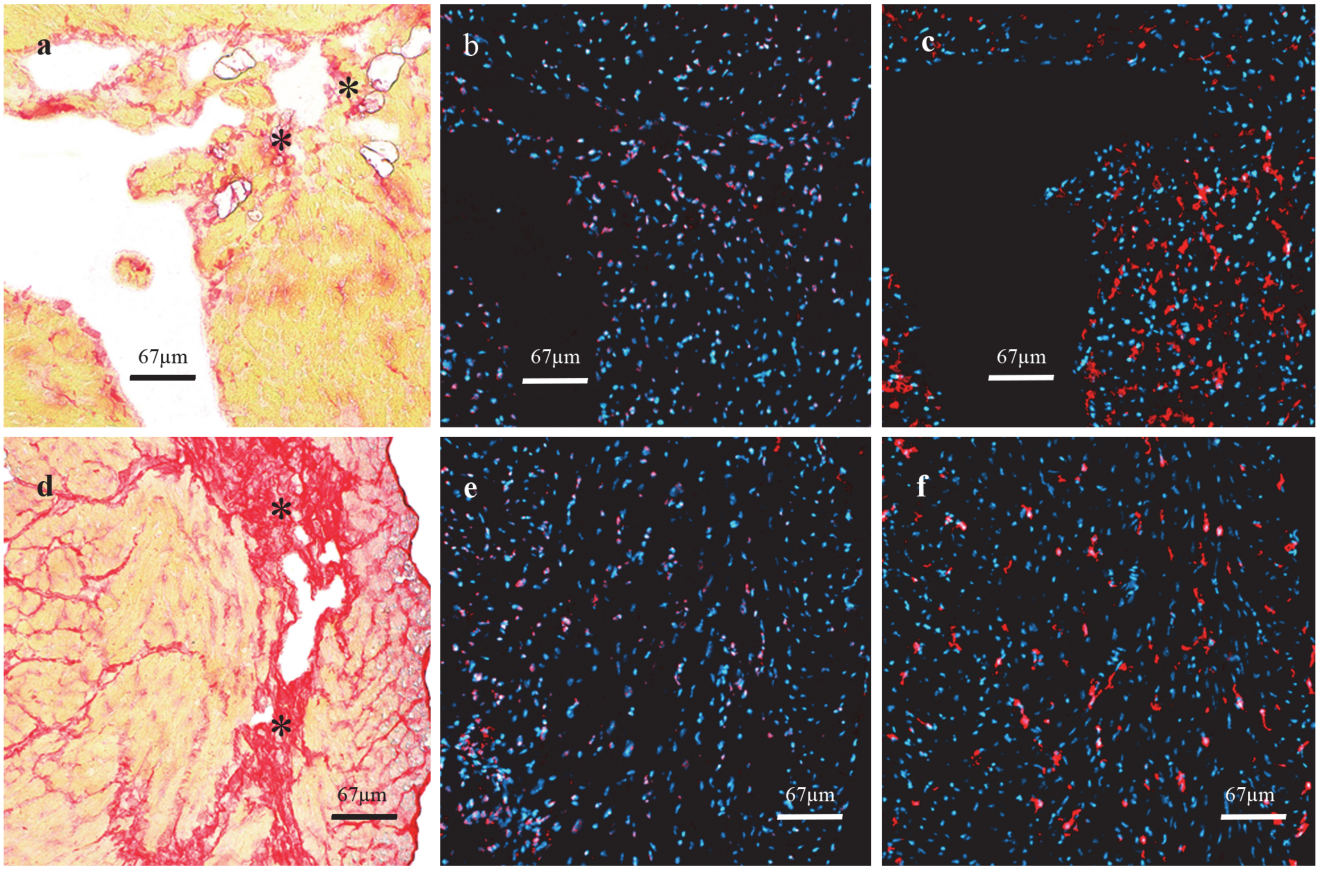

Fig 5. Histological findings. Upper row: one animal of the 5-month group (0.5 Gy, LDR) showed disseminated collageneous stripes (*) after sirius red staining **a)**, corresponding accumulations of **b)** CD45^{+ve} and **c)** collagen IV^{+ve} cells. Lower row: in four animals of the 8-month group (2 Gy, LDR) similar findings were found: broad collagenous scars **d)** going along with CD45^{+ve} **e)** and collagen IV^{+ve} **f)** immunoreactivity.

doi:10.1371/journal.pone.0119661.g005

LDR are presented in separate diagrams (Fig. 6A-D) as relative mean ± SEM compared to control samples of sham-irradiated animals. Values for INF and IL-10 before and after irradiation were too low to be measured accurately. Therefore, they are not presented in the figure. Nevertheless, it can be stated, that no massive increase of both markers took place as levels stayed around lowest standard concentration (2.5 pg/ml). Exposure at LDR compared to sham irradiation led to a significant increase of proinflammatory cytokines (TNFα, MCP-1) and to a prothrombotic response with enhanced fibrinogen levels in animals examined 3 months after irradiation. Also KC levels tended to increase, whereas levels of the soluble adhesion marker sICAM decreased significantly. A local enhancement of TGFβ was found at 0.05 Gy only (Fig. 6A). At the later time point, 6 months after exposure, TNFα and MCP-1 levels dropped back to control, whereas elevated fibrinogen levels persisted and sVCAM/sICAM levels increased at the highest dose (2 Gy). Responses were observed already at very low doses (0.025 Gy for sICAM and fibrinogen) but more parameters changed at doses of 0.05 Gy and higher (Fig. 6B). Similarly to LDR, exposure at HDR significantly enhanced TNFα and MCP-1 levels 3 months after irradiation (Fig. 6C). Levels of soluble adhesion molecules (mainly sVCAM) were significantly reduced 3 months, but also 6 months, after exposure. In contrast to LDR, fibrinogen levels remained unchanged after exposure at HDR, and expression of the anti-inflammatory marker TGFβ decreased at both time points. TGFβ measurements, however, showed a high level of variance, sometimes resulting in trends rather than in significances. In addition, a

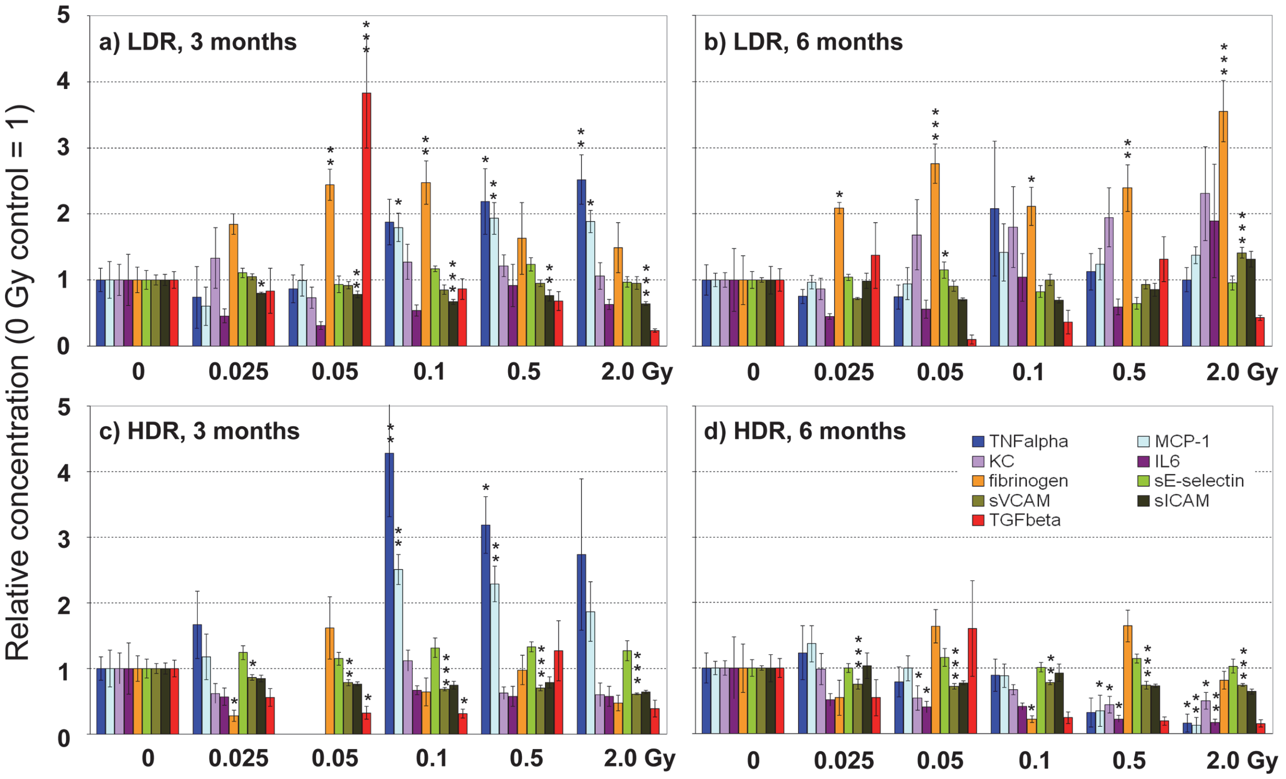

Fig 6. Dose-dependent effects on plasma markers. Concentrations of plasma markers were measured 3 (**a, c**) and 6 (**b, d**) months after irradiation at the indicated doses at low dose rate (LDR, **a,b**) and high dose rate (HDR, **c,d**). Values are presented as mean ± SEM, relative to sham-irradiated control (0 Gy = 1). Missing bars in Fig. 6C are due to analytical failure. *Asterisks* indicate p-values ≤ 0.05: *, p ≤ 0.01: **, p ≤ 0.001: *** compared to control.

doi:10.1371/journal.pone.0119661.g006

concomitant decrease of proinflammatory cytokines (TNFα, MCP-1, KC and IL6) was observed 6 months after irradiation (Fig. 6D). Kendall tau correlation analysis demonstrated a significant overall correlation of proinflammatory cytokines (TNFα, KC, IL6 and MCP-1) in all groups ($p < 0.05$). These markers increased in the LDR group simultaneously 3 and 6

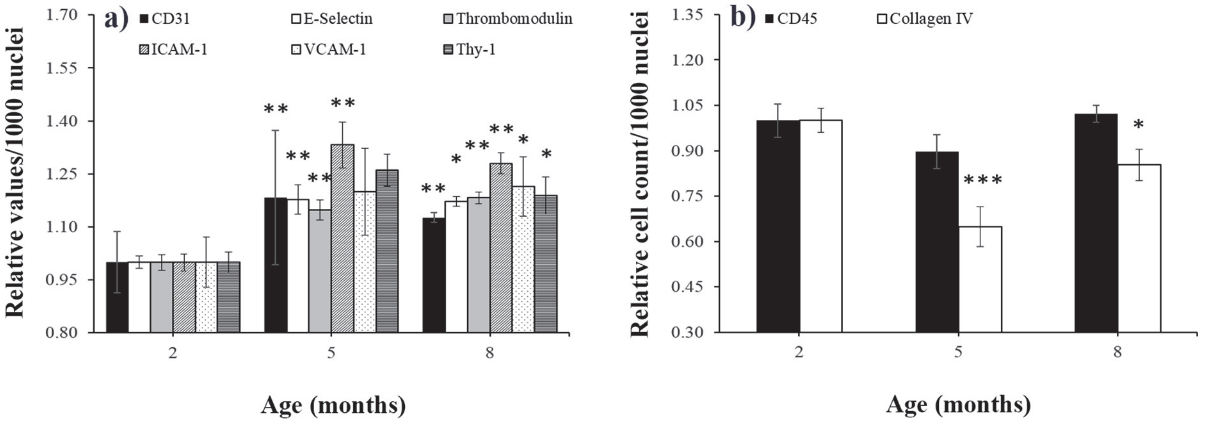

Fig 7. Age-specific effects on tissue markers in nonirradiated mice. The expression of **a**) CD31, E-selectin, thrombomodulin, ICAM-1, VCAM-1, Thy-1 and of **b**) CD45 and collagen IV^{+ve} cells is presented in sham-irradiated 0 Gy control mice at 2, 5, and 8 months of age. The relative number of specifically stained cells per 1000 nuclei around the left ventricle of the heart is given (mice at 2 months = 1, mean ± SEM, n = 8). Asterisks indicate p-values ≤ 0.05: *, p ≤ 0.01: **, p ≤ 0.001: *** compared to the 2-month group.

doi:10.1371/journal.pone.0119661.g007

months after exposure, whereas in the HDR group elevated values were found only 3 months after irradiation. Six months after irradiation at HDR a significant decrease of these markers might indicate a counter-regulatory response.

From the panel of 11 candidate markers investigated here, TNFα, MCP-1, sICAM, sVCAM, TGFβ and fibrinogen, turned out to be the most sensitive markers to evaluate systemic plasma responses towards low-dose irradiation.

Age-specific effects

Analysis of basal marker expression in non- or sham irradiated animals revealed a significant age effect. Data are given as the mean of all subgroups (sham irradiation with high dose rate [HDR] and low dose rate [LDR]) ± SEM, presented as absolute values per 1000 nuclei. Analysis for endothelial proteins (CD31, E-selectin, thrombomodulin, ICAM-1, VCAM-1 and Thy-1) revealed a significant age-dependent increase at 5 and 8 months compared with animals at 2 month of age (Fig. 7A). For CD45 and collagen IV^{+ve} cells, adverse findings could be observed: cell counts decreased to minimum levels at 5 months and increased at 8 months back to levels at 2 months of age. These findings reached significance levels for collagen IV^{+ve} cells at 5 and 8 months (Fig. 7B). Some proinflammatory cytokines (KC, MCP-1) and fibrinogen behaved similarly and increased significantly at the age of 5 and 8 months compared with 2-month-old animals. In contrast, soluble adhesion molecules (sVCAM, sICAM and sE-selectin) levels slightly decreased with age. Only IL6, TGF and TNF levels did not change (data not shown).

Discussion

High radiation doses are known to induce cardiac microvascular damage and are often associated with inflammatory and fibrotic changes [15,48–50]. The effects of low doses on the vasculature however, are non-linear and can not be extrapolated from high doses. High-dose irradiation entails both protective and harmful effects but very low doses (0.025–0.05 Gy), given at a low dose rate, were shown to be protective [13]. Different dose rates are affiliated with partly contrary effects on atherosclerotic lesion growth and severity. Moreover, the time point of applied irradiation has a large influence at the disease progression [51].

In this study we examined late effects of low-dose irradiation with specific emphasis on heart microvascular damage, using an atherosclerosis-prone $ApoE^{-/-}$ mouse model. We demonstrate here, that low doses from 0.025 Gy may induce significant changes on the expression of inflammatory and thrombotic markers. In tissues, we found these effects to be independent of the dose rate applied and similar at 3 and 6 months after irradiation, except for collagen IV. In contrast, plasma markers showed more dose rate- and time-dependent variation.

Local effects of exposure on heart tissue

The significant increase of **$CD31^{+ve}$** cells indicates a gain of capillaries after irradiation with 2 Gy. This might be regarded as a compensatory mechanism since replacement cell proliferation can be induced when cell depletion reaches critical levels [52]. However high IR doses (16 Gy) lead to irreversible damage and decrease the microvascular density [50]. We found no changes in capillary size e.g. by endothelial swelling and CD31 expression/cell remained stable. Reports on human umbilical vein ECs [53], dermal microvascular ECs [54], and human lung microvascular ECs [55] showing significant increases in CD31 expression and/or size 3–21 days after 5–10 Gy irradiation imply that these parameters may be also affected but at higher doses than used here.

The dose-dependent changes in cell counts of the endothelial cell markers **CD31, thrombomodulin, and E-selectin** were highly similar, and show nearly identical curve shapes.

Therefore, we assume that the increase of thrombomodulin and E-selectin^{+ve} cells at 2 Gy is mainly attributable to the increment of capillaries. The lack of an irradiation effect on mFI (protein expression) or mean area (cell size) supports the notion, that irradiation at these doses has no long-term effect on these markers. Other groups observed an early response resulting in increased expression of E-selectin, a few hours after irradiation with low to high doses (0.5–10 Gy) [56–58]. Underpinning the short-term dynamic of this marker, unchanged levels of E-selectin have been reported in irradiated HUVEC (2–10 Gy) evident 1–10 days after irradiation [59].

For thrombomodulin, increased immunoreactivity and release as an acute response, followed by a downregulation at later time points, has been reported after high dose IR. In HUVECs IR-induced damage was associated with an acute release of thrombomodulin at 24 h after irradiation at 12.5 Gy, followed by a reduced capacity of the cells to produce and release thrombomodulin 6 days after exposure [27]. Studies in human and rat intestines revealed a significant reduction in the microvascular thrombomodulin expression 2–56 months after high-dose (33.6–55 Gy) IR [60,61]. Depending on the genetic background, differential results have been shown in ApoE$^{-/-}$ *versus* C57BL/6J mice [18]. For low doses, as applied in our study, no valid data are available so far.

In this study, no significant changes of **ICAM-1** expression in the heart microvasculature were detected, and only a slight reduction of ICAM at very low doses was seen. Others found increased ICAM-1 levels at earlier time points (3 h - 10 days) after irradiation with moderate to high doses (5–25 Gy) in human endothelial cells *in vitro* [54,59,62], in human skin organ cultures [56] and *in vivo* in rat liver [63]. Apparently, ICAM levels normalize within a few weeks after IR, though VCAM-1 and leukocyte adherence may remain elevated [64]. In accordance, we found small increases of **VCAM-1** expression on the endocardial endothelium at 0.5 Gy, reaching significant levels at 2 Gy. This is in line with our previous findings in A. saphena, showing increased VCAM immunoreactivity 3 months after 2 Gy IR [65] and suggests a late inflammatory response at higher doses. Irradiation-induced alterations of VCAM-1 expression have also been described at early time points (several days) in numerous tissues [54,56,59,63,64] indicating acute and chronic response of this marker after IR.

Cardiac fibrosis is one of the late sequelae of high-dose IR. Thereby, fibrosis is often preceded by inflammation and capillary injury, followed by an increased synthesis of extracellular matrix (ECM) including collagens [66,67]. **Collagen IV**, produced by interstitial fibroblasts and cardiomyocytes, is a major component of basement membranes [34,68]. It accumulates during early fibrotic processes in numerous organs [32,33,69] and initiates collagenous replacement. Publications about expression of collagen IV alpha 3 in the heart vary widely and certainly species-specific differences exist [69,70]. In contrast to the well known localization of collagen 1/2 around the myocyte fibers, we found collagen 4 to be located mostly in small deposits in close association with endothelial cells. In accordance, collagen IV alpha 3 chains have been described to directly interact with endothelial surface receptors, namely integrins [71], playing a role in endothelial cell migration during angiogenesis.

Data about collagen IV alterations after IR are rare. We could demonstrate for the first time significantly enhanced collagen IV deposits 3 months after IR with very low dose levels (0.025 Gy), which returned to control levels at 6 months. This time course follows reports about early increased collagen deposits at 20 but not at 40 weeks after high dose IR (16 Gy) in mice [72]. Similarly, Miller *et al.* reported elevated collagen IV levels 4–5 months following irradiation of up to 14 Gy [35]. Collagen IV deposits were, in our case, not necessarily linked with leukocyte invasion. Only in a few animals with massive fibrotic strands in sirius red staining were increased leukocyte counts also detected. In contrast, at very low doses, leukocyte numbers were slightly diminished with a minimum at 0.025 Gy but returned to normal at 2 Gy. These

findings support the assumption that very low dose IR primarily induces anti-inflammatory effects, in contrast to higher doses [13,51].

Thy-1, is species-dependently expressed on fibroblasts [73] and endothelial cells [74], but also on T-cells and neurons [75,76]. Thy-1 induction seems to play an important role in the acute and chronic inflammatory response and has been shown to mediate leukocyte adherence to microvascular cells in the lung [74]. The here observed dose-dependent decrease of Thy-1^{+ve} cell counts 3 and 6 months after irradiation exposure, therefore, might be interpreted as a late downregulation of endothelial inflammatory response at low doses (significant changes were found at 0.025 up to 0.5 Gy). However, loss of Thy-1 is associated with a profibrotic phenotype in lung fibroblasts [77]. In this respect, together with the elevated collagen IV levels, Thy-1 decrease might also indicate fibroblast activation. Activated fibroblasts can trigger numerous processes like fibrosis and myocardial hypertrophy [78] and might contribute to a compensatory response of the heart towards low-dose IR.

Systemic effects of exposure on plasma markers

The radiation-induced local anti-inflammatory effects described above, were paralleled by an increase of plasma markers, like fibrinogen, TNFα and MCP-1, indicating a long term systemic prothrombotic and proinflammatory response, already at very low doses (from 0.025 Gy onwards). The parallel decrease of TGFβ, an anti-inflammatory marker, might further enhance the cytokine release. Such effects have been widely described before, but only for higher doses and mostly after short observation periods [37]. Long-term effects (120 days) were found after 10 Gy TBI exposures leading to significantly enhanced fibrinogen levels [45]. Van der Meeren *et al*. reported increases of IL6 and KC after TBI in C57BL6/J mice exposed to lethal doses of gamma rays 10 to 18 days after irradiation. Cytokines can be released by endothelial cells as well as by leukocytes. Circulating adhesion molecules, however, are shed from their counterparts mainly located on endothelial cells and, therefore, indicate more clearly a vascular response. In contrast to cytokines and fibrinogen, sICAM and sVCAM levels decreased after irradiation exposure of doses below 2 Gy. This indicates an anti-inflammatory vascular effect and goes along with decreases of ICAM and Thy-1 presented in the heart immunofluorescence stainings. The data fit well also into our recent findings in the same mouse cohort, where we showed, that low-dose exposures up to 0.5 Gy result in a significant decrease of the number and average aortic lesion area 3 months after irradiation exerting protective, anti-inflammatory effects [13].To date, however, we can not exclude that soluble adhesion molecules increase acutely but transiently and that the late-time decrease found here, represents a counter-regulatory/compensatory effect. Although the increase of soluble adhesion markers in inflammatory processes and during irradiation is well described [41–43], long-term responses are rarely reported so far. Stewart *et al.* measured sVCAM and sICAM levels in ApoE$^{-/-}$ mice 28 weeks after single radiation doses of 14 Gy to the neck and found no differences between irradiated and nonirradiated groups, indicating that even high doses of local irradiation do not translate into long-term systemic effects [16].

Our investigation of a broad panel of markers clearly shows, that only some inflammatory markers stay elevated after longer periods of time. From 11 candidate plasma markers only six, TNFα, MCP-1, sICAM, sVCAM, TGFβ, and fibrinogen, showed changes 3 or 6 months after irradiation. Effects were non-linear with doses up to 0.5 Gy. Especially acute markers as IL-6 and sE-selectin might only transiently increase, as suggested by others [41]. To get a clearer picture of the short- and long-time responses of these markers, we will evaluate short term effects of low-dose irradiation in a similar experimental setting in the next future.

Age-related effects

The age-dependent significant increases of basal inflammatory marker expression between 2 and 5–8 months in ApoE$^{-/-}$ mice (Fig. 7) is possibly a result of the enhanced LDL-cholesterol plasma levels, which induces, among other processes, the expression of leukocyte adhesion molecules in endothelial cells [79]. We could also demonstrate significant age-related changes of plasma markers in ApoE$^{-/-}$ mice, which is in line with recent studies showing increases in circulating inflammatory markers in the elderly [80,81]. An evaluation of systemic markers of inflammation in atomic bomb survivors revealed collective effects of radiation and aging, possibly enhancing the persistent inflammatory status in this group [36].

Conclusion

We have shown that low irradiation doses (from 0.025 Gy) induce local and systemic changes in an inflammatory-prone genotype. Thereby, high dose rates exerted more pronounced effects than low dose rates, although the general pattern of response was similar. Screening of 19 inflammation-related candidates revealed 11 markers (MCP-1, TNF, TGF, (s)ICAM-1, (s)VCAM-1, fibrinogen, Thy1, collagen IV, CD31) to be of high value to identify even small late effects of low-dose irradiation. Our findings in ApoE$^{-/-}$mice suggest that low radiation doses (0.025 – 0.5 Gy) induce adaptive late responses, such as increases of capillary density and changes of collagen IV and Thy-1 tissue levels, may reflect compensatory regulations in the heart. Only at the highest dose (2 Gy), the enhancement of VCAM-1 expression on the endocardium may indicates a proinflammatory response. On the other hand, a reduction of inflammatory vascular markers along with decreases of soluble adhesion molecules in the blood at 0.025–0.5 Gy underpins the anti-inflammatory characteristics of low-dose irradiation. The systemic increase of TNF and MCP-1 at 0.025–0.5 Gy might indicate a differential response of markers, additionally derived by leukocytes.

The new insights into the multiple low-dose radiation-induced inflammatory/thrombotic changes, may give important implications for the anti-inflammatory radiotherapy. The systemic changes of plasma markers may facilitate an individual evaluation of radiation-related, late adverse effects and even initiate adequate intervention strategies. If these effects are relevant in metabolically normal people or only in such with metabolic risk factors, such as elevated cholesterol levels, remains to be determined.

Acknowledgments

We are grateful to Tobias Polte and colleagues for technical support with the fluorescence microscope and analysis software. We thank Gabriela Aust and fellow workers for technical support with tissue cutting. Many thanks also to Katrin Bauer and Heike Knaack for their help with cytokine detection by cytometric bead array and ELISA.

Author Contributions

Conceived and designed the experiments: REJM NDP SCW GH. Performed the experiments: DM M. Barclay HW M. Bugden AG. Analyzed the data: DM AG MS MK. Contributed reagents/materials/analysis tools: REJM NDP SCW GH MS MK. Wrote the paper: DM AG.

References

1. Takahashi I, Abbott RD, Ohshita T, Takahashi T, Ozasa K, Akahoshi M, et al. A prospective follow-up study of the association of radiation exposure with fatal and non-fatal stroke among atomic bomb survivors in Hiroshima and Nagasaki (1980–2003). BMJ Open 2012; 2: e000654. doi: 10.1136/bmjopen-2011-000654 PMID: 22307102

2. Baker JE, Moulder JE, Hopewell JW. Radiation as a risk factor for cardiovascular disease. Antioxid Redox Signal. 2011; 15: 1945–1956. doi: 10.1089/ars.2010.3742 PMID: 21091078
3. Sakata R, Grant EJ, Ozasa K. Long-term follow-up of atomic bomb survivors. Maturitas. 2012; 72: 99–103. doi: 10.1016/j.maturitas.2012.02.009 PMID: 22440534
4. Jurcut R, Savu O, Giusca S, Deleanu D, Ciudin R, Ginghina C. Between Scylla and Charybdis: long-term cardiovascular complications after radiotherapy for Hodgkin's lymphoma. Hellenic J Cardiol. 2009; 50: 538–543. PMID: 19942568
5. Galper SL, Yu JB, Mauch PM, Strasser JF, Silver B, Lacasce A, et al. Clinically significant cardiac disease in patients with Hodgkin lymphoma treated with mediastinal irradiation. Blood. 2011; 117: 412–418. doi: 10.1182/blood-2010-06-291328 PMID: 20858859
6. Howe GR, Zablotska LB, Fix JJ, Egel J, Buchanan J. Analysis of the mortality experience amongst U.S. nuclear power industry workers after chronic low-dose exposure to ionizing radiation. Radiat Res. 2004; 162: 517–526. PMID: 15624306
7. Gyenes G. Radiation-induced ischemic heart disease in breast cancer—a review. Acta Oncol. 1998; 37: 241–246. PMID: 9677094
8. Lee PJ, Mallik R. Cardiovascular effects of radiation therapy: practical approach to radiation therapy-induced heart disease. Cardiol Rev. 2005; 13: 80–86. PMID: 15705258
9. Heidenreich PA, Kapoor JR. Radiation induced heart disease: systemic disorders in heart disease. Heart. 2009; 95: 252–258. doi: 10.1136/hrt.2008.149088 PMID: 19144884
10. Fajardo LF, Berthrong M. Vascular lesions following radiation. Pathol Annu. 1988; 23 Pt 1: 297–330. PMID: 3387138
11. Stewart JR, Fajardo LF. Radiation-induced heart disease: an update. Prog Cardiovasc Dis. 1984; 27: 173–194. PMID: 6387801
12. Boerma M, Hauer-Jensen M. Preclinical research into basic mechanisms of radiation-induced heart disease. Cardiol Res Pract. 2010; 2011: 858262. doi: 10.4061/2011/858262 PMID: 20953374
13. Mitchel RE, Hasu M, Bugden M, Wyatt H, Little MP, Gola A, et al. Low-dose radiation exposure and atherosclerosis in ApoE(-)/(-) mice. Radiat Res. 2011; 175: 665–676. doi: 10.1667/RR2176.1 PMID: 21375359
14. Meir KS, Leitersdorf E. Atherosclerosis in the apolipoprotein-E-deficient mouse: a decade of progress. Arterioscler Thromb Vasc Biol. 2004; 24: 1006–1014. PMID: 15087308
15. Patties I, Haagen J, Dorr W, Hildebrandt G, Glasow A. Late inflammatory and thrombotic changes in irradiated hearts of C57BL/6 wild-type and atherosclerosis-prone ApoE-deficient mice. Strahlenther Onkol. 2015; 2:172–179 PMID: 25200359
16. Hoving S, Heeneman S, Gijbels MJ, Te Poele JA, Visser N, Cleutjens J, et al. Irradiation induces different inflammatory and thrombotic responses in carotid arteries of wildtype C57BL/6J and atherosclerosis-prone ApoE(-/-) mice. Radiother Oncol. 2012; 105: 365–370. doi: 10.1016/j.radonc.2012.11.001 PMID: 23245647
17. Burch GE, Sohal RS, Sun SC, Miller GC, Colcolough HL. Effects of radiation on the human heart. An electron microscopic study. Arch Intern Med. 1968; 121: 230–234. PMID: 5642742
18. Weintraub NL, Jones WK, Manka D. Understanding radiation-induced vascular disease. J Am Coll Cardiol. 2010; 55: 1237–1239. doi: 10.1016/j.jacc.2009.11.053 PMID: 20298931
19. Walther M, Kaffenberger W, Van BD. Influence of clinically used antioxidants on radiation-induced expression of intercellular cell adhesion molecule-1 on HUVEC. Int J Radiat Biol. 1999; 75: 1317–1325. PMID: 10549609
20. Quarmby S, Kumar P, Kumar S. Radiation-induced normal tissue injury: role of adhesion molecules in leukocyte-endothelial cell interactions. Int J Cancer. 1999; 82: 385–395. PMID: 10399956
21. Baluna RG, Eng TY, Thomas CR. Adhesion molecules in radiotherapy. Radiat Res. 2006; 166: 819–831. PMID: 17149971
22. Wetzel A, Chavakis T, Preissner KT, Sticherling M, Haustein UF, Anderegg U, et al. Human Thy-1 (CD90) on activated endothelial cells is a counterreceptor for the leukocyte integrin Mac-1 (CD11b/CD18). J Immunol. 2004; 172: 3850–3859. PMID: 15004192
23. Wetzel A, Wetzig T, Haustein UF, Sticherling M, Anderegg U, Simon JC, et al. Increased neutrophil adherence in psoriasis: role of the human endothelial cell receptor Thy-1 (CD90). J Invest Dermatol. 2006; 126: 441–452. PMID: 16374458
24. Hauer-Jensen M, Fink LM, Wang J. Radiation injury and the protein C pathway. Crit Care Med. 2004; 32: S325–S330. PMID: 15118539
25. Zhou Q, Zhao Y, Li P, Bai X, Ruan C. Thrombomodulin as a marker of radiation-induced endothelial cell injury. Radiat Res. 1992; 131: 285–289. PMID: 1332109

26. Ross CC, MacLeod SL, Plaxco JR, Froude JW, Fink LM, Wang J, et al. Inactivation of thrombomodulin by ionizing radiation in a cell-free system: possible implications for radiation responses in vascular endothelium. Radiat Res. 2008; 169: 408–416. doi: 10.1667/RR1148.1 PMID: 18363428

27. Van de Wouwer M, Conway EM. Novel functions of thrombomodulin in inflammation. Crit Care Med. 2004; 32: S254–S261. PMID: 15118527

28. Zhou QS, Zhao YM, Xu CS, Yu ZY, Yao DY, Gao Y, et al. Increase in plasma thrombomodulin and decrease in plasma von Willebrand factor after regular radiotherapy of patients with cancer. Thromb Res. 1992; 68: 109–118. PMID: 1335614

29. Abreu-Velez AM, Howard MS. Collagen IV in Normal Skin and in Pathological Processes. N Am J Med Sci. 2012; 4: 1–8. doi: 10.4103/1947-2714.92892 PMID: 22393540

30. Zeisberg M, Bonner G, Maeshima Y, Colorado P, Muller GA, Strutz F, et al. Renal fibrosis: collagen composition and assembly regulates epithelial-mesenchymal transdifferentiation. Am J Pathol. 2001; 159: 1313–1321. PMID: 11583959

31. Gomez-Reino JJ, Sandberg M, Carreira PE, Vuorio E. Expression of types I, III and IV collagen genes in fibrotic skin and nerve lesions of toxic oil syndrome patients. Clin Exp Immunol. 1993; 93: 103–107. PMID: 8391940

32. Eghbali M, Blumenfeld OO, Seifter S, Buttrick PM, Leinwand LA, Robinson TF, et al. Localization of types I, III and IV collagen mRNAs in rat heart cells by in situ hybridization. J Mol Cell Cardiol 1989; 21: 103–113. PMID: 2534136

33. Miller GG, Kenning JM, Dawson DT. Radiation-induced changes in collagen isotypes I, III, and IV in the lung of LAF1 mouse: effects of time, dose, and WR-2721. Radiat Res. 1988; 115: 515–532. PMID: 2845466

34. Hayashi T, Morishita Y, Khattree R, Misumi M, Sasaki K, Hayashi I, et al. Evaluation of systemic markers of inflammation in atomic-bomb survivors with special reference to radiation and age effects. FASEB J. 2012; 26: 4765–4773. doi: 10.1096/fj.12-215228 PMID: 22872680

35. Schultz-Hector S, Trott KR. Radiation-induced cardiovascular diseases: is the epidemiologic evidence compatible with the radiobiologic data? Int J Radiat Oncol Biol Phys. 2007; 67: 10–18. PMID: 17189062

36. Roedel F, Kley N, Beuscher HU, Hildebrandt G, Keilholz L, Kern P, et al. Anti-inflammatory effect of low-dose X-irradiation and the involvement of a TGF-beta1-induced down-regulation of leukocyte/endothelial cell adhesion. Int J Radiat Biol. 2002; 78: 711–719. PMID: 12194755

37. Kern PM, Keilholz L, Forster C, Hallmann R, Herrmann M, Seegenschmiedt MH. Low-dose radiotherapy selectively reduces adhesion of peripheral blood mononuclear cells to endothelium in vitro. Radiother Oncol. 2000; 54: 273–282 PMID: 10738086

38. Hildebrandt G, Seed MP, Freemantle CN, Alam CA, Colville-Nash PR, Trott KR. Mechanisms of the anti-inflammatory activity of low-dose radiation therapy. Int J Radiat Biol. 1998; 74: 367–378. PMID: 9737539

39. Matsuda Y, Hara J, Osugi Y, Tokimasa S, Fujisaki H, Takai K, et al. Serum levels of soluble adhesion molecules in stem cell transplantation-related complications. Bone Marrow Transplant. 2001; 27: 977–982. PMID: 11436109

40. Ishii Y, Kitamura S. Soluble intercellular adhesion molecule-1 as an early detection marker for radiation pneumonitis. Eur Respir J. 1999; 13: 733–738. PMID: 10362032

41. Blankenberg S, Rupprecht HJ, Bickel C, Peetz D, Hafner G, Tiret L, et al. Circulating cell adhesion molecules and death in patients with coronary artery disease. Circulation. 2001; 104: 1336–1342. PMID: 11560847

42. Corrado E, Rizzo M, Coppola G, Fattouch K, Novo G, Marturana I, et al. An update on the role of markers of inflammation in atherosclerosis. J Atheroscler Thromb. 2010; 17: 1–11. PMID: 20032572

43. Baker JE, Fish BL, Su J, Haworth ST, Strande JL, Komorowski RA, Mirgrino RQ, et al. 10 Gy total body irradiation increases risk of coronary sclerosis, degeneration of heart structure and function in a rat model. Int J Radiat Biol. 2009; 85(12): 1089–1100. doi: 10.3109/09553000903264473 PMID: 19995235

44. Mulrooney DA, Ness KK, Solovey A, Hebbel RP, Neaton JD, Peterson BA, et al. Pilot study of vascular health in survivors of Hodgkin lymphoma. Pediatr Blood Cancer. 2012; 59: 285–289. doi: 10.1002/pbc.24082 PMID: 22457206

45. Stec JJ, Silbershatz H, Tofler GH, Matheney TH, Sutherland P, Lipinska I, et al. Association of fibrinogen with cardiovascular risk factors and cardiovascular disease in the Framingham Offspring Population. Circulation. 2000; 102: 1634–1638. PMID: 11015340

46. Fajardo LF, Stewart JR. Pathogenesis of radiation-induced myocardial fibrosis. Lab Invest. 1973; 29: 244–257. PMID: 4724850

47. Lauk S, Kiszel Z, Buschmann J, Trott KR. Radiation-induced heart disease in rats. Int J Radiat Oncol Biol Phys. 1985; 11: 801–808. PMID: 3980275
48. Gabriels K, Hoving S, Seemann I, Visser NL, Gijbels MJ, Pol JF, et al. Local heart irradiation of ApoE (-/-) mice induces microvascular and endocardial damage and accelerates coronary atherosclerosis. Radiother Oncol. 2012; 105: 358–364. doi: 10.1016/j.radonc.2012.08.002 PMID: 22959484
49. Seemann I, Gabriels K, Visser NL, Hoving S, Te Poele JA, Pol JF, et al. Irradiation induced modest changes in murine cardiac function despite progressive structural damage to the myocardium and microvasculature. Radiother Oncol. 2012; 103: 143–150. doi: 10.1016/j.radonc.2011.10.011 PMID: 22112779
50. Mitchel RE, Hasu M, Bugden M, Wyatt H, Hildebrandt G, Chen Y, et al. Low-dose radiation exposure and protection against atherosclerosis in ApoE(-/-) mice: the influence of P53 heterozygosity. Radiat Res. 2013; 179: 190–199. doi: 10.1667/RR3140.1 PMID: 23289388
51. Lauk S, Trott KR. Endothelial cell proliferation in the rat heart following local heart irradiation. Int J Radiat Biol. 1990; 57: 1017–1030. PMID: 1970990
52. Quarmby S, Kumar P, Wang J, Macro JA, Hutchinson JJ, Hunter RD, et al. Irradiation induces upregulation of CD31 in human endothelial cells. Arterioscler Thromb Vasc Biol. 1999; 19: 588–597. PMID: 10073961
53. Quarmby S, Hunter RD, Kumar S. Irradiation induced expression of CD31, ICAM-1 and VCAM-1 in human microvascular endothelial cells. Anticancer Res. 2000; 20: 3375–3381. PMID: 11131637
54. Gaugler MH, Vereycken-Holler V, Squiban C, Aigueperse J. PECAM-1 (CD31) is required for interactions of platelets with endothelial cells after irradiation. J Thromb Haemost. 2004; 2: 2020–2026. PMID: 15550034
55. Heckmann M, Douwes K, Peter R, Degitz K. Vascular activation of adhesion molecule mRNA and cell surface expression by ionizing radiation. Exp Cell Res. 1998; 238: 148–154. PMID: 9457067
56. Hildebrandt G, Maggiorella L, Rodel F, Rodel V, Willis D, Trott KR. Mononuclear cell adhesion and cell adhesion molecule liberation after X-irradiation of activated endothelial cells in vitro. Int J Radiat Biol. 2002; 78: 315–325. PMID: 12020443
57. Hallahan D, Clark ET, Kuchibhotla J, Gewertz BL, Collins T. E-selectin gene induction by ionizing radiation is independent of cytokine induction. Biochem Biophys Res Commun. 1995; 217: 784–795. PMID: 8554599
58. Gaugler MH, Squiban C, van der Meeren A, Bertho JM, Vandamme M, Mouthon MA, et al. Late and persistent up-regulation of intercellular adhesion molecule-1 (ICAM-1) expression by ionizing radiation in human endothelial cells in vitro. Int J Radiat Biol. 1997; 72: 201–209. PMID: 9269313
59. Wang J, Zheng H, Ou X, Fink LM, Hauer-Jensen M. Deficiency of microvascular thrombomodulin and up-regulation of protease-activated receptor-1 in irradiated rat intestine: possible link between endothelial dysfunction and chronic radiation fibrosis. Am J Pathol. 2002; 160: 2063–2072. PMID: 12057911
60. Richter KK, Fink LM, Hughes BM, Sung CC, Hauer-Jensen M. Is the loss of endothelial thrombomodulin involved in the mechanism of chronicity in late radiation enteropathy? Radiother Oncol. 1997; 44: 65–71. PMID: 9288860
61. Hallahan D, Kuchibhotla J, Wyble C. (Cell adhesion molecules mediate radiation-induced leukocyte adhesion to the vascular endothelium. Cancer Res. 1996; 56: 5150–5155. PMID: 8912850
62. Moriconi F, Malik I, Ahmad G, Dudas J, Rave-Frank M, Vorwerk H, et al. Effect of irradiation on gene expression of rat liver adhesion molecules: in vivo and in vitro studies. Strahlenther Onkol. 2009; 185: 460–468. doi: 10.1007/s00066-009-1964-1 PMID: 19714308
63. Molla M, Gironella M, Miquel R, Tovar V, Engel P, Biete A, et al. Relative roles of ICAM-1 and VCAM-1 in the pathogenesis of experimental radiation-induced intestinal inflammation. Int J Radiat Oncol Biol Phys. 2003; 57: 264–273. PMID: 12909242
64. Patties I, Habelt B, Rosin B, Dorr W, Hildebrandt G, Glasow A. Late effects of local irradiation on the expression of inflammatory markers in the Arteria saphena of C57BL/6 wild-type and ApoE-knockout mice. Radiat Environ Biophys. 2013; 53: 117–124. doi: 10.1007/s00411-013-0492-7 PMID: 24071970
65. Fajardo LF, Stewart JR. Capillary injury preceding radiation-induced myocardial fibrosis. Radiology. 1971; 101: 429–433. PMID: 5114783
66. Stewart JR, Fajardo LF, Gillette SM, Constine LS. Radiation injury to the heart. Int J Radiat Oncol Biol Phys. 1995; 31: 1205–1211. PMID: 7713783
67. Miner JH, Sanes JR. Collagen IV alpha 3, alpha 4, and alpha 5 chains in rodent basal laminae: sequence, distribution, association with laminins, and developmental switches. J Cell Biol. 1994; 127: 879–891. PMID: 7962065
68. Yamanishi A, Kusachi S, Nakahama M, Ninomiya Y, Watanabe T, Kumashiro H, et al. Sequential changes in the localization of the type IV collagen alpha chain in the infarct zone: immunohistochemical

study of experimental myocardial infarction in the rat. Pathol Res Pract. 1998; 194: 413–422. PMID: 9689650

69. Leinonen A, Mariyama M, Mochizuki T, Tryggvason K, Reeders ST. Complete primary structure of the human type IV collagen alpha 4(IV) chain. Comparison with structure and expression of the other alpha (IV) chains. J Biol Chem. 1994; 269: 26172–26177. PMID: 7523402

70. Mariyama M, Leinonen A, Mochizuki T, Tryggvason K, Reeders ST. Complete primary structure of the human alpha 3(IV) collagen chain. Coexpression of the alpha 3(IV) and alpha 4(IV) collagen chains in human tissues. J Biol Chem. 1994; 269: 23013–23017. PMID: 8083201

71. Seemann I, Te Poele JA, Luikinga SJ, Hoving S, Stewart FA. Endoglin haplo-insufficiency modifies the inflammatory response in irradiated mouse hearts without affecting structural and mircovascular changes. PLoS One. 2013; 8: e68922. doi: 10.1371/journal.pone.0068922 PMID: 23894375

72. Hudon-David F, Bouzeghrane F, Couture P, Thibault G. Thy-1 expression by cardiac fibroblasts: lack of association with myofibroblast contractile markers. J Mol Cell Cardiol. 2007; 42: 991–1000. PMID: 17395197

73. Schubert K, Polte T, Bonisch U, Schader S, Holtappels R, Hildebrandt G, et al. Thy-1 (CD90) regulates the extravasation of leukocytes during inflammation. Eur J Immunol. 2011; 41: 645–656. doi: 10.1002/eji.201041117 PMID: 21264853

74. Haeryfar SM, Hoskin DW. Thy-1: more than a mouse pan-T cell marker. J Immunol. 2004; 173: 3581–3588. PMID: 15356100

75. Jurisic G, Iolyeva M, Proulx ST, Halin C, Detmar M. Thymus cell antigen 1 (Thy1, CD90) is expressed by lymphatic vessels and mediates cell adhesion to lymphatic endothelium. Exp Cell Res. 2010; 316: 2982–2992. doi: 10.1016/j.yexcr.2010.06.013 PMID: 20599951

76. Ramirez G, Hagood JS, Sanders Y, Ramirez R, Becerril C, Segura L, et al. Absence of Thy-1 results in TGF-beta induced MMP-9 expression and confers a profibrotic phenotype to human lung fibroblasts. Lab Invest. 2011; 91: 1206–1218. doi: 10.1038/labinvest.2011.80 PMID: 21577212

77. Ieda M, Tsuchihashi T, Ivey KN, Ross RS, Hong TT, Shaw RM, et al. Cardiac fibroblasts regulate myocardial proliferation through beta1 integrin signaling. Dev Cell. 2009; 16: 233–244. doi: 10.1016/j.devcel.2008.12.007 PMID: 19217425

78. Stewart FA, Heeneman S, Te Poele J, Kruse J, Russell NS, Gijbels M, et al. Ionizing radiation accelerates the development of atherosclerotic lesions in ApoE-/- mice and predisposes to an inflammatory plaque phenotype prone to hemorrhage. Am J Pathol. 2006; 168: 649–658. PMID: 16436678

79. Mertens A, Holvoet P. Oxidized LDL and HDL: antagonists in atherothrombosis. FASEB J. 2001; 15: 2073–2084. PMID: 11641234

80. Miles EA, Rees D, Banerjee T, Cazzola R, Lewis S, Wood R, et al. Age-related increases in circulating inflammatory markers in men are independent of BMI, blood pressure and blood lipid concentrations. Atherosclerosis 2008; 196: 298–305. PMID: 17118371

81. Krabbe KS, Pedersen M, Bruunsgaard H. Inflammatory mediators in the elderly. Exp Gerontol. 2004; 39: 687–699. PMID: 15130663
